# Characterization and mechanism of simultaneous degradation of aflatoxin B_1_ and zearalenone by an edible fungus of *Agrocybe cylindracea* GC-Ac2

**DOI:** 10.3389/fmicb.2024.1292824

**Published:** 2024-02-13

**Authors:** Changying Guo, Lixia Fan, Qingqing Yang, Mingxiao Ning, Bingchun Zhang, Xianfeng Ren

**Affiliations:** ^1^Institute of Quality Standard and Testing Technology for Agro-products, Shandong Academy of Agricultural Sciences, Jinan, China; ^2^Shandong Provincial Key Laboratory of Test Technology on Food Quality and Safety, Jinan, China; ^3^School of Agricultural Engineering and Food Science, Shandong University of Technology, Zibo, China

**Keywords:** edible fungi, aflatoxin B1, zearalenone, manganese peroxidase, simultaneous degradation

## Abstract

Contamination with multiple mycotoxins is a major issue for global food safety and trade. This study focused on the degradation of aflatoxin B_1_ (AFB_1_) and zearalenone (ZEN) by 8 types of edible fungi belonging to 6 species, inclulding *Agaricus bisporus*, *Agrocybe cylindracea*, *Cyclocybe cylindracea*, *Cyclocybe aegerita*, *Hypsizygus marmoreus* and *Lentinula edodes.* Among these fungi, *Agrocybe cylindracea* strain GC-Ac2 was shown to be the most efficient in the degradation of AFB_1_ and ZEN. Under optimal degradation conditions (pH 6.0 and 37.4°C for 37.9 h), the degradation rate of both AFB_1_ and ZEN reached over 96%. Through the analysis of functional detoxification components, it was found that the removal of AFB_1_ and ZEN was primarily degraded by the culture supernatant of the fungus. The culture supernatant exhibited a maximum manganese peroxidase (MnP) activity of 2.37 U/mL. Interestingly, *Agrocybe cylindracea* strain GC-Ac2 also showed the capability to degrade other mycotoxins in laboratory-scale mushroom substrates, including 15A-deoxynivalenol, fumonisin B_1_, B_2_, B_3_, T-2 toxin, ochratoxin A, and sterigmatocystin. The mechanism of degradation of these mycotoxins was speculated to be catalyzed by a complex enzyme system, which include MnP and other ligninolytic enzymes. It is worth noting that *Agrocybe cylindracea* can degrade multiple mycotoxins and produce MnP, which is a novel and significant discovery. These results suggest that this candidate strain and its enzyme system are expected to become valuable biomaterials for the simultaneous degradation of multiple mycotoxins.

## Introduction

Mycotoxins are indeed a major issue in food and feed safety. They are produced by various fungi such as *Aspergillus, Fusarium, Penicillium*, and *Alternaria* (Escriva et al., 2017). These fungi can contaminate agricultural products both before and after harvest, leading to the presence of mycotoxins in food and feed. Aflatoxins (AFTs), fumonisins (FBs), zearalenone (ZEN), deoxynivalenol (DON), ochratoxin A (OTA), T-2 toxin (T-2), and sterigmatocystin (ST) are among the most common mycotoxins found in food and feed samples (Schatzmayr and Streit, 2013; Tolosa et al., 2021). These mycotoxins can adversely affect human and animal health, including the development of cancer, tumors, reproductive system disorders, and neural tube defects (Wild and Gong, 2010; Zain, 2011). Agricultural products are widely contaminated with mycotoxins, which has significant impacts on global health, economy and international trade. According to the Food and Agriculture Organization of the United Nations (FAO), approximately 25% of global agricultural products is contaminated with mycotoxins. This contamination results in substantial food losses, estimated at nearly 1 billion tons annually (Wang and Xie, 2020). Therefore, mitigating mycotoxin contamination is crucial to ensuring the safety and quality of food and feed.

In fact, to mitigate mycotoxin contamination in food and feed, a variety of strategies have been explored, including chemical, physical and biological approaches. Chemical methods such as ozonation, acid or alkali hydrolysis, and ammoniation have been utilized to reduce mycotoxin levels (Luo et al., 2013). Physical techniques like physical adsorption, ultrafiltration percolation, γ-radiation treatment and microwave heating have also shown some effectiveness (Pankaj et al., 2018). However, these traditional chemical and physical approaches do have certain limitations. They can be inefficient, cause nutritional losses, leave residual toxicity, and even alter the properties of processed food (Peng et al., 2018). In recent years, alternative methods such as cold plasma (Shi et al., 2017; Wu et al., 2021) and photoirradiation (Fanelli et al., 2016) have been proposed for food processing. However, further research is still needed to fully understand the degradation products, specific mechanisms of action, and the impact of these techniques on the nutritional content and sensory qualities of processed foods. Therefore, while progress has been made in developing strategies to reduce mycotoxins, research and exploration are still ongoing to find more effective, safe and reliable methods.

Biological methods, such as microbial adsorption/binding and degradation, have received widespread attention as promising alternatives for reducing mycotoxins in food and feed. These methods offer the advantages of strong specificity, high efficiency and environmental friendliness (Zhu et al., 2016; Guan et al., 2021). A variety of microorganisms, including lactic acid bacteria, yeasts and fungal conidia, have shown the ability to effectively adsorb mycotoxins such as AFTs, ZEN, DON, OTA and FBs (Luo et al., 2018). However, the application of microbial adsorption is currently limited due to incomplete removal and a lack of information on its potential impact on nutritional quality and the potential toxicity of the resulting by-products (Haque et al., 2020). In addition to adsorption, many bacteria, fungi, yeasts and their enzymes have demonstrated the capability to degrade mycotoxins in laboratory settings and real food matrices (Adebo et al., 2017; Loi et al., 2017; Bi et al., 2018; Guo et al., 2020; Loi et al., 2020). However, it is important to consider the introduction of new contaminants that may arise during microbial fermentation. In this context, edible fungi have huge application potential due to their edible, enjoyable and nutritious characteristics. Edible fungi can serve a dual purpose, both degrading mycotoxins and providing additional nutritional value to food or feed products. Further research and development is needed to explore the efficacy, safety, and feasibility of using edible fungi to reduce mycotoxins.

In this study, a strain of the edible fungus *Agrocybe cylindracea* called GC-Ac2 was firstly identified with the ability to degrade multiple mycotoxins *in vitro* and in practical substrates. More strikingly, *Agrocybe cylindracea* strain GC-Ac2 produced highly active manganese peroxidase (MnP) in its culture supernatant. The study also demonstrated that a complex system of enzymes secreted by *Agrocybe cylindracea* GC-Ac2 was responsible for the degradation of mycotoxins, specifically AFB_1_ and ZEN. The findings of this study provide valuable insights into the potential use of *Agrocybe cylindracea* and its enzymes as valuable biomaterials for effective detoxification of multiple mycotoxins.

## Materials and methods

### Edible fungus strains, chemicals and culture media

The eight strains used in this study are all edible white rot fungi (Table 1). Strains CGMCC5.2 and CGMCC5.1 were purchased from the China General Microbiological Culture Collection Center (CGMCC),^1^ Beijing, China. The isolates of GC-Ac2, GC-Cc74, GC-Hm06, GC-Hm23, GC-Hm59 and GC-Le95 are strains commercially developed for mushroom production, and were obtained from a local farm specializing in mushroom cultivation in Shandong province, China. These isolates were molecularly characterized by analyzing the sequences of the internal transcribed spacers ITS-1 and ITS-2 of nuclear rDNA. These strains were cultured on potato dextrose agar (PDA) slants and used as sources of inocula for subsequent cultures.

**Table 1:** Strains of the edible white rot fungi used in this study.

Species	Strains	Geographical origin	Source
*Agaricus bisporus*	CGMCC5.2	China	Bought from CGMCC
*Cyclocybe aegerita*	CGMCC5.1	China	Bought from CGMCC
*Agrocybe cylindracea*	GC-Ac2	Shandong, China	Isolated from Mushroom substrate
*Cyclocybe cylindracea*	GC-Cc74	Shandong, China	Isolated from Mushroom substrate
*Hypsizygus marmoreus*	GC-Hm06	Shandong, China	Isolated from Mushroom substrate
	GC-Hm23	Shandong, China	
	GC-Hm59	Shandong, China	
*Lentinula edodes*	GC-Le95	Shandong, China	Isolated from Mushroom substrate

Mycotoxin standards were purchased from Sigma-Aldrich (St. Louis, MO, United States). Water was purified by a Milli-Q purification system (Millipore, Bedford, MA, United States). All other chemicals and reagents purchased from local chemical stores were of analytical grade or higher.

### Removal of AFB_1_ and ZEN by eight strains

Eight strains were tested for their ability to remove AFB_1_ and ZEN in liquid cultures of potato dextrose broth (PDB). Assays were carried out in 12-well plates (Costar Inc., Cambridge, MA, United States). Each well was filled with 2 mL of PDB supplemented with AFB_1_ and ZEN standards at approximately 100 ng/mL and 200 ng/mL, respectively, and then inoculated with a 6 mm-diameter mycelial disk from a 15-day-old culture of a strain on PDA. After 10 days of incubation at 28°C in the dark, 1 mL culture medium was taken from each well, filtered through 0.22-μm filter membrane, and analyzed by ultra-performance liquid chromatography with tandem mass spectroscopy (UPLC-MS/MS) to determine AFB_1_ and ZEN. Wells filled with PDB supplemented with AFB_1_ and ZEN standards but not inoculated with strains were used as controls. The removal rate was calculated by the formula of F_1_.


$$F_{1}\;\left(\%\right)=\left[\left(C_{i}-C_{f}\right)/C_{i}\right]\times 100$$

where C_i_ is the concentration of AFB_1_ or ZEN in the control wells and *C_f_* is the concentration of AFB_1_ or ZEN in the testing wells.

### Joint inhibitory effects of AFB_1_ and ZEN on the growth of eight strains

The joint inhibitory effects of AFB_1_ and ZEN on the growth of eight strains were studied in PDA media supplemented with approximately 3 μg/mL AFB_1_ plus 5 μg/mL ZEN. The PDA media were prepared and sterilized. After cooling but not solidifying, the media were supplemented with AFB_1_ and ZEN standards to the desired concentration, thoroughly mixed and poured into 9 cm-diameter petri dishes. Each culture dish was inoculated with a 6 mm-diameter mycelial disk of one isolate and then incubated in the dark at 28°C for 10 days. Five replicates were prepared for each tested isolate. Controls were inoculated with mycelial disks, but not supplemented with mycotoxins. Growth of the strains was assessed by colony diameter measured under a dissecting microscope. The percentage of growth inhibition was calculated by the formula of F_2_.


$$F_{2}\;\left(\%\right)=\left[\left(D_{i}-D_{f}\right)/D_{i}\right]\times 100$$

where D_i_ is the diameter of the fungus colony grown on control agar, and D_f_ is the diameter of the fungus colony grown on agar supplemented with AFB_1_ and ZEN.

### Determination of MnP and laccase activities in culture supernatant of eight strains

PDB (100 mL) was prepared in a flask, inoculated with ten 6 mm-diameter mycelial disks of one strain and incubated at 28°C with constant shaking (175 rpm). After 10 days of incubation, 2 mL of culture supernatant was collected from each of the triplicated flasks and filtered through 0.22-μm filter membrane for measurement of MnP and laccase activities.

In the presence of Mn^2+^, 2,6-dimethoxyphenol (2,6-DMP, ε = 49,600/M/cm) can be oxidized by MnP. The activity of MnP can be determined by monitoring the oxidation of 2, 6-DMP at 470 nm with a ultraviolet and visible (UV–VIS) spectrophotometer. The reaction mixture (1.2 mL) contained 180 μL of culture supernatant, 1 mM MnSO_4_, 1 mM of 2,6-DMP and 0.1 mM H_2_O_2_ in 50 mM sodium malonate buffer. One unit (1 U) of MnP activity was defined as the amount of enzyme that oxidizes 1 nmol of 2,6-DMP per minute under standard assay conditions. Laccase activity was measured photometrically by monitoring the oxidation of 2,2′-azino-bis (3-ethylbenzothiazoline-6-sulphonic acid), ABTS (ε = 36,000/M/cm), at 420 nm. Reactions were performed in buffer containing 100 mM sodium acetate, 2 mM ABTS and 150 μL of culture supernatant in a final volume of 1 mL (pH 4.5 and 25°C). After ABTS oxidation, absorbance increased at 420 nm. 1 U of laccase activity was defined as the amount of enzyme which produced 1 μmol of product per minute under assay conditions.

### Pattern analysis of AFB_1_ and ZEN removal by *Agrocybe cylindracea* GC-Ac2

According to the above experimental results, *Agrocybe cylindracea* GC-Ac2 was considered as the most promising strain, and was therefore used for further research. To determine the functional components of GC-Ac2, its culture supernatant, intracellular component and mycelia were analyzed for their effect on AFB_1_ and ZEN removal ability, according to the assay described by Li et al. (2018) with some modifications. Ten 6 mm-mycelial disks of the strain were added to PDB (100 mL) and incubated at 28°C with constant shaking (175 rpm). After 10 days of incubation, the mixture was filtered through the filter paper and then through a 0.22-μm filter membrane. The filtrate was used as culture supernatant. Meanwhile, mycelia pellets were collected after filtration through filter paper, washed twice with phosphate-buffered saline (PBS), and drained with filter paper. Then, the mycelia were immediately ground in liquid nitrogen, re-suspended with PBS (5 mL) and sonicated (400 W, 25 kHz) in an ice bath for 30 min, and then centrifuged at 15,000 *g* for 10 min. The suspension was used as an intracellular component. The culture supernatant and intracellular components were supplemented with AFB_1_ and ZEN standards to approximately 500 ng/mL, stored under static conditions at 37°C for 48 h, and analyzed for concentration by UPLC-MS/MS. Additionally, MnP activity of culture supernatant and intracellular components was measured using the method described in the previous section.

Studies have reported that mycotoxins absorbed by cell walls could be released back into solution through elution with polar solvents (Haidukowski et al., 2019). Therefore, desorption experiments were conducted to obtain the adsorption ratios as described in our previous paper (Yue et al., 2022). AFB_1_ and ZEN were added to PDB medium (100 mL) in the flask to a final concentration of approximately 500 ng/mL. Then, ten 6-mm-diameter GC-Ac2 plugs were added to the flask and incubated for 10 days at 28°C with constant shaking (175 rpm). The mycelium pellets were collected, washed gently twice with PBS, and then treated with 25 mL acetonitrile with constant shaking (200 rpm) for 1 h to elute AFB_1_ and ZEN adsorbed by the pellets. The solvents were then recovered for AFB_1_ and ZEN determination by UPLC-MS/MS.

### Optimizing the conditions for simultaneous degradation of AFB_1_ and ZEN in the culture supernatant of *Agrocybe cylindracea* GC-Ac2

The culture supernatant was prepared as described in the previous section. AFB_1_ and ZEN standards were added to the culture supernatant to approximately 100 ng/mL and 200 ng/mL, respectively, and incubated under static conditions in the dark at pH 5.8 and 37°C. Triplicates were collected after 0, 2, 12, 24, 48 and 72 h. The culture supernatant was adjusted to pH values of 2, 4, 6, 7, 8 and 9 with HCl (12 mol) and NaOH (2 mol). Culture supernatants at different pH values were supplemented with AFB_1_ and ZEN standards to approximately 100 ng/mL and 200 ng/mL, and then incubated under static conditions in the dark at 37°C for 48 h. The degradation activity of the culture supernatant (pH 5.8) was also measured in temperature range from 4°C to 121°C to determine the optimal temperature. Culture supernatants treated at different temperatures were incubated under static conditions in the dark for 48 h, and then analyzed by UPLC-MS/MS to determine AFB_1_ and ZEN. The same aliquots of PDB treated in the same manner were used as controls.

### Box-Behnken design and response surface methodology

The appropriate single-factor range for AFB_1_ and ZEN degradation by GC-Ac2 culture supernatant was determined through single-factor test in the previous section. To investigate the optimal conditions that can be used to obtain the optimal simultaneous degradation efficiency, BB design and RSM were employed to study the three most important factors affecting simultaneous degradation. Reaction time, temperature and pH were used as three effective factors (A, B and C, respectively), and 3 levels (−1, 0 and 1) were taken to find the optimal conditions (Supplementary Table S1). Since simultaneous degradation conditions were more needed in practical applications, the average degradation rates of AFB_1_ and ZEN were selected as the response values for response surface optimization. The experimental design matrix and response values are shown in Supplementary Table S2, where the data were analyzed using Design-Expert 12.0.3 software.

### Toxicity analysis of AFB_1_ and ZEN degradation products in the culture supernatant of *Agrocybe cylindracea* GC-Ac2

PDB (100 mL) was prepared in a flask, supplemented with AFB_1_ and ZEN standards to a final concentration of 500 ng/mL each, inoculated with ten 6 mm-diameter mycelial disks of GC-Ac2 and incubated at 28°C with constant shaking (175 rpm). After 10 days of incubation, the culture supernatant containing AFB_1_ and ZEN degradation products was collected from the flask and used for the toxicity analysis. Toxicity was evaluated by a micronucleus assay in mouse bone marrow erythrocytes. The test was carried out in accordance with China National Standards GBZ/T 240.11–2011 with some modification. Mice (half male and half female) were intragastrically fed with degradation product solution (0.2 mL/10 g.BW) twice (with an interval of 24 h each time). After the last feeding, bone marrow samples were collected for smears. The smears were fixed in methanol for 10 min. After drying, smears were stained with freshly prepared Giemsa for 10 min, and then immediately washed with PBS. The number of polychromatic erythrocytes containing micronucleus was then observed and counted using a microscope. Sterilized water was used as a negative control and cyclophosphamide (40 mg/kg.BW) was used as a positive control.

### MnP activity dynamics and AFB_1_/ZEN removal dynamics in the culture supernatant of *Agrocybe cylindracea* GC-Ac2

PDB (100 mL) was prepared in a flask, supplemented with AFB_1_ and ZEN standards to approximately 100 ng/mL and 200 ng/mL, respectively, inoculated with ten 6 mm-diameter mycelial disks of GC-Ac2 and incubated at 28°C with constant shaking (175 rpm). After 2, 4, 6, 8, 10, 13 and 15 days of incubation, 2 mL of supernatant was collected from each one of the triplicated flasks, 1 mL of the supernatant was analyzed by UPLC-MS/MS to determine AFB_1_ and ZEN, and 1 mL was used to measure MnP activity. Simultaneously, the control flask was supplemented with 100 ng/mL AFB_1_ and 200 ng/mL ZEN but without the inoculation with GC-Ac2. Mathematical correlations between MnP activity and AFB_1_/ZEN removal were obtained using the model equations of Boltzmann and OriginPro9.0 (OriginLab Corporation, Northampton, US).

### Effects of heat, proteinase K + SDS, EDTA, NaN_3_, Mn^2+^ (10 mM), H_2_O_2_ (5 mM) and Mn^2+^ (1 mM) + H_2_O_2_ (0.1 mM) + sodium malonate (50 mM) on the activity of the culture supernatant *Agrocybe cylindracea* GC-Ac2 to degrade AFB_1_ and ZEN

Culture supernatant of *Agrocybe cylindracea* GC-Ac2 was prepared as described above, and following treatments were performed: autoclaving at 121°C for 30 min; treatment with 1 mg/mL proteinase K plus 1% (w/v) sodium dodecyl sulfonate (SDS) at 55°C for 2 h; treatment with 10 mM ethylene diamine tetraacetic acid (EDTA) at 37°C for 2 h; treatment with 0.1% (w/v) sodium azide (NaN_3_) at 37°C for 2 h; treatment with 10 mM Mn^2+^ from MnSO_4_ at 37°C for 2 h; treatment with 5 mM H_2_O_2_ at 37°C for 2 h; treatment with 1 mM Mn^2+^ + 0.1 mM H_2_O_2_ + 50 mM sodium malonate at 37°C for 2 h. PDB was treated in the same manner and used as a control. After treatments, the culture supernatant and control were supplemented with AFB_1_ and ZEN to final concentrations of 100 ng/mL and 200 ng/mL, respectively, stored under static conditions in the dark at 37°C for 24 h, and then analyzed by UPLC-MS/MS to determine AFB_1_ and ZEN concentrations.

### Culture of *Agrocybe cylindracea* GC-Ac2 in mushroom substrate containing naturally contaminated maize, wheat and peanut meal

The strain of *Agrocybe cylindracea* GC-Ac2 was cultured on a substrate similar to that used to produce mushroom spawns, but containing naturally contaminated maize, wheat and peanut meal. The substrate contained 60% maize naturally contaminated with ZEN, FBs and T-2, 10% wheat naturally contaminated with DONs and OTA, 10% peanut meal naturally contaminated with AFB_1_ and ST, 13% cottonseed hull, 2% gypsum powder, 4.9% magnesium sulfate and 0.1% potassium dihydrogen phosphate. The substrates (60 g) were added into a vessel, thoroughly mixed, and 100 mL of tap water was added to achieve a moisture content of approximately 65% (w/w). The mixture was autoclaved at 121°C for 120 min and then inoculated with 20 mycelial disks of strain GC-Ac2 and cultured for 20 days. Uninoculated vessels containing contaminated substrate were used as controls. After incubation, the substrate was dried and ground into powder ready for preparation. The powder was then added into 50 mL of ACN/water/formic acid (84/15.9/0.1, v/v/v) solution, shaken for 10 min, and sonicated for 30 min. The mixture was centrifuged at 2,600 *g* for 15 min, and then the centrifugate was concentrated using a nitrogen sweeping system. The concentrate was re-suspended and analyzed by UPLC-MS/MS to determine multiple mycotoxins.

### Determination of AFB_1_ and ZEN using UPLC-MS/MS

Mycotoxins were determined using a UPLC-MS/MS platform. Chromatographic separations were performed on a Waters Acquity-System (Milford, MA, United States). The column used for LC separations was a 100 mm × 2.1 mm i.d., 1.7-μm, Acquity UPLC BEH C18, equipped with an AcquityUPLC column in-line filter (0.2-μm). Sample and column temperatures were set at 15°C and 35°C, respectively. The mobile phase consisted of eluent A (deionized water containing 0.1% formic acid) and eluent B (methanol). A binary gradient with a flow rate of 0.3 mL/min was programmed.

MS/MS analyses were performed on a triple quadrupole mass spectrometer (QTRAP 5500 with an electrospray ionization (ESI) source, AB Sciex, Framingham, MA, United States) in a positive MRM mode. The mass parameters were as follows: 5.5 kV ion spray voltage, 20 psi curtain gas pressure, 60 psi pressure for the nebulizer (gas 1) and turbo (gas 2) gases, and 550°C turbo heater temperature. For targeted quantitative analysis of mycotoxins, including ion confirmation using one quantifier and one qualifier transition, the transitions monitored (corresponding collision energy) were as follows: AFB_1_: 313.3 → 285.1 (30 V); 313.3 → 241.1 (48 V), the declustering potential voltage was set to 100 V; ZEN: 317.0 → 131.0 (39 V); 317.0 → 175.0 (34 V), the declustering potential voltage was set to 110 V. With this condition, the retention time of AFB_1_ and ZEN was about 4.5 min and 6.3 min, respectively. The method has a limit of quantification (LOQ) of 0.2 ng/mL based on a signal-to-noise ratio of 10:1.

### Statistical analysis

Data were analyzed by one-way analysis of variance (ANOVA) and Tukey–Kramer multiple comparison test. Statistical analyses were performed using the GraphPadInstat 3.0 software (GraphPad Software, San Diego, CA). Figures were drawn using OriginPro9.0 software. When *p* < 0.001, the difference is considered extremely significant; when *p* < 0. 01, the difference is highly significant; when *p* < 0. 05, the difference is significant; when *p* > 0. 05, the difference is insignificant.

## Results and discussions

### Removal of AFB_1_ and ZEN by eight strains

In addition to AFB_1_, ZEN is a common mycotoxin contaminant in food and feed. Therefore, we set out to investigate whether strains could remove multiple mycotoxins simultaneously such as ZEN and AFB_1_. The removal efficiency of AFB_1_ and ZEN in PDB by eight strains of edible fungi is shown in Table 2. These strains belong to species including *Agaricus bisporus, Agrocybe cylindracea, Cyclocybe aegerita, Hypsizygus marmoreus and Lentinula edodes*. These strains had different capacities to remove AFB_1_ and ZEN. After 10 days of incubation in PDB, seven isolates except *Agaricus bisporus* CGMCC5.2 could remove more than 70% of AFB_1_ and ZEN. The removal rates of AFB_1_ and ZEN by the strains with higher efficiency, *Agrocybe cylindracea* GC-Ac2 and *Lentinula edodes* GC-Le95, were both higher than 90.0%. Among them, the removal rates of AFB_1_ and ZEN by *Agrocybe cylindracea* strain GC-Ac2 were 100 and 94.4%, respectively.

**Table 2:** Removal rates of AFB_1_ and ZEN by the white rot fungi cultured in PDB supplemented with 100 ng/mL AFB_1_, 200 ng/mL ZEN after being cultured at 28°C in the dark for 10 days.

Species	Strains	AFB_1_ ± SD (ng/mL)		Removal (%)	ZEN ± SD (ng/mL)		Removal (%)
Control		99.8 ± 1.7	A		192.7 ± 3.6	A	
*Agaricus bisporus*	CGMCC5.2	79.0 ± 2.8	B	20.8	175.8 ± 3.2	B	8.2
*Cyclocybe aegerita*	CGMCC5.1	15.6 ± 0.7	D	84.3	38.1 ± 1.3	D	80.2
*Agrocybe cylindracea*	**GC-Ac2**	**nd**	**E**	**100**	**10.8 ± 1.5**	**E**	**94.4**
*Cyclocybe cylindracea*	GC-Cc74	15.0 ± 0.7	D	85.0	36.4 ± 1.0	D	81.1
*Hypsizygus marmoreus*	GC-Hm06	24.3 ± 1.5	C	75.7	29.5 ± 2.1	D	84.7
	GC-Hm23	16.2 ± 1.6	D	83.7	32.2 ± 2.2	D	83.3
	GC-Hm59	18.7 ± 1.7	CD	81.3	52.4 ± 1.5	C	72.8
*Lentinula edodes*	GC-Le95	2.8 ± 0.6	E	97.1	13.6 ± 1.0	E	92.9

Data represent the mean values of AFB_1_ and ZEN in 3 replicated cultures ± SD. Percent removal of Data represent the means ± SD of AFB_1_ and ZEN in 3 replicated cultures. The calculation formula for the removal rate of AFB_1_ and ZEN is: F (%) = [(C_i_ − C_f_)/ C_i_] × 100, where C_i_ is the concentration of AFB_1_ in the uninoculated control and C_f_ is the concentration of AFB_1_ and ZEN in the fungal culture at a given time. Different capital letters are statistically different (*p* < 0.001) according to Tukey–Kramer Multiple Comparison test.

Mycotoxin contamination is a global issue with significant impacts on human and animal health as well as the world economy and international trade (Terzi et al., 2014; Mitchell et al., 2016). Controlling contamination at multiple points of cultivation, harvesting, transportation, storage and processing is necessary, but detoxification is particularly important once contamination is uncontrolled or control points fail (Adebo et al., 2016). The use of edible fungi to detoxify mycotoxins has attracted attention. In addition to their detoxifying properties, edible fungi also have the advantage of providing nutrients. However, it is important to note that not all edible fungi have the ability to detoxify mycotoxins, and their effectiveness may vary depending on the specific mycotoxin and fungal species. Some specific species of mushrooms, such as *Pleurotus ostreatus*, *Pleurotus eryngii*, *Lentinula edodes*, *Agaricus bisporus*, *Hericium erinaceus*, etc. (Kunze et al., 2017; Lou et al., 2023), have been found to be able to effectively degrade or transform mycotoxins. Compared with these edible fungi, *Agrocybe cylindracea* strain GC-Ac2 demonstrates the highest removal efficiency for AFB_1_ and ZEN and has broad prospects.

### Joint inhibitory effects of AFB_1_ and ZEN on the growth of eight strains

**Figure 1:**
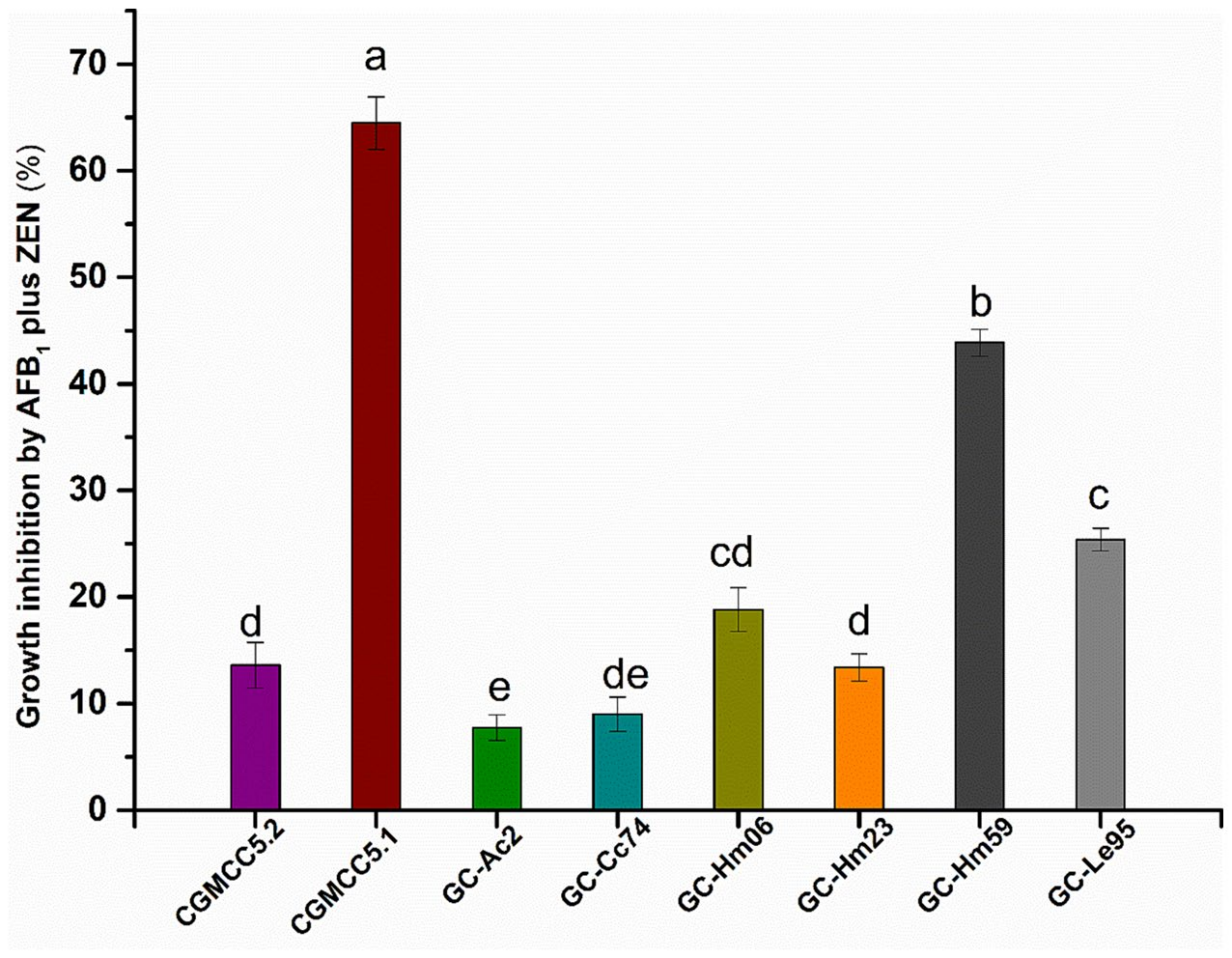
Joint inhibitory effects of AFB_1_ (3 μg/mL) and ZEN (5 μg/mL) on growth of the eight strains including *Agaricus bisporus* CGMCC5.2, *Cyclocybe aegerita* CGMCC5.1, *Agrocybe cylindracea* GC-Ac2, *Cyclocybe cylindracea* GC-Cc74, *Hypsizygus marmoreus* GC-Hm06, GC-Hm23 and GC-Hm59, and *Lentinula edodes* GC-Le95. Bars with different letters are significantly different for *p* < 0.001 (Tukey–Kramer multiple comparison test).

The growth inhibition results of AFB_1_ and ZEN on the eight strains are shown in Figure 1. Eight strains showed different sensitivities to mycotoxins when exposed to 3 μg/mL of AFB_1_ and 5 μg/mL of ZEN. *Cyclocybe aegerita* CGMCC5.1 was the most sensitive strain, with a growth inhibition rate of 64.5% by AFB_1_ and ZEN. In contrast, AFB_1_ and ZEN had almost no effect on the growth of *Agrocybe cylindracea* GC-Ac2 and *Cyclocybe cylindracea* GC-Cc74, with inhibition rates of 7.8 and 9.0%, respectively. Furthermore, the tolerance of *Agrocybe cylindracea* strain GC-Ac2 and *Cyclocybe cylindracea* GC-Cc74 to AFB_1_ (3 μg/mL) and ZEN (5 μg/mL) indicates their potential for wider application and practical use in real substrates (Yue et al., 2022).

### Activities of MnP and laccase in culture supernatants of eight strains

**Figure 2:**
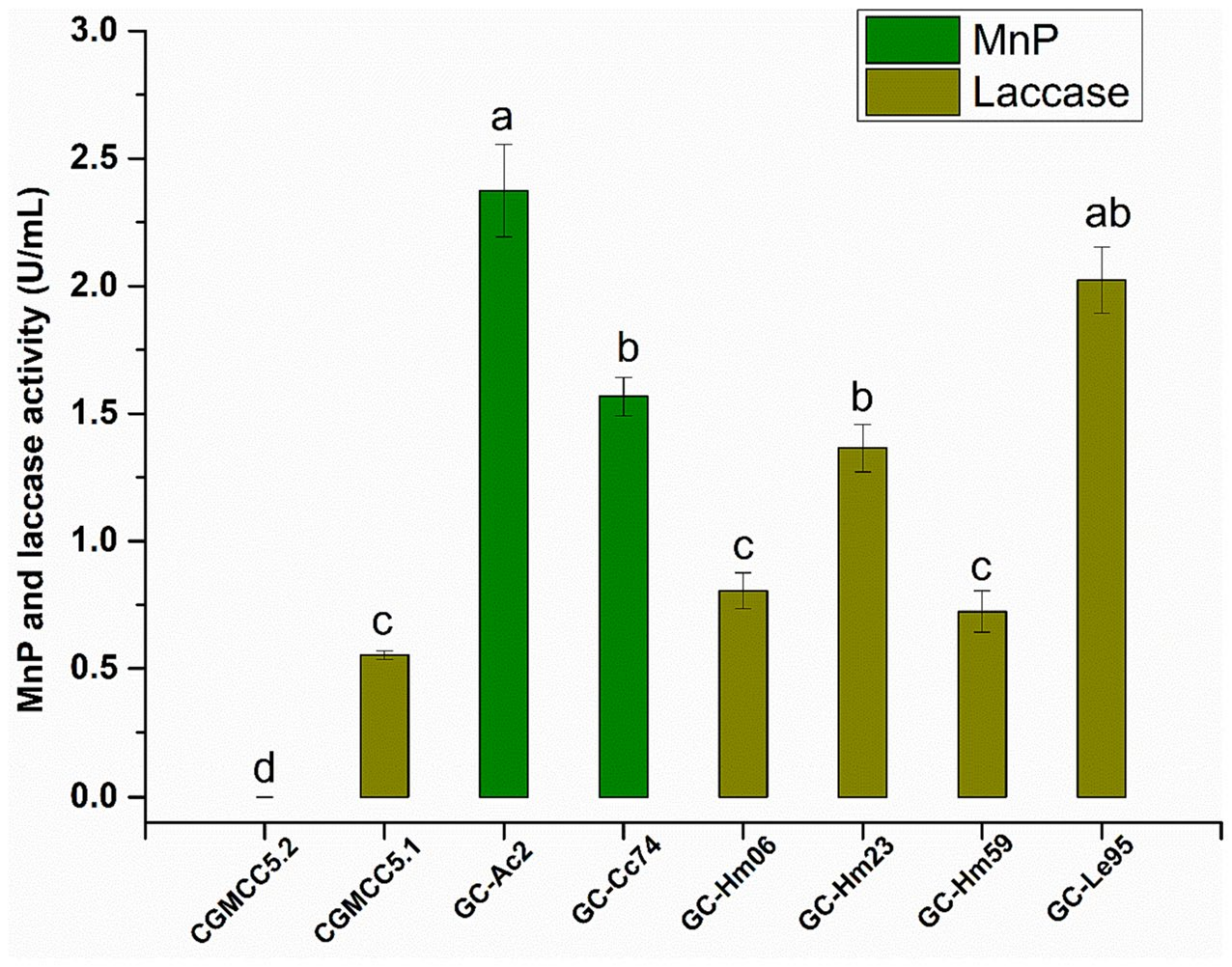
The activity of MnP and laccase in the culture supernatant of the eight strains (*Agaricus bisporus* CGMCC5.2, *Cyclocybe aegerita* CGMCC5.1, *Agrocybe cylindracea* GC-Ac2, *Cyclocybe cylindracea* GC-Cc74, *Hypsizygus marmoreus* GC-Hm06, GC-Hm23 and GC-Hm59, and *Lentinula edodes* GC-Le95) after cultured in PDB for 10 days. Bars with different letters are significantly different for *p* < 0.001 (Tukey–Kramer multiple comparison test).

The MnP and laccase activities secreted into the culture supernatant by the eight strains are shown in Figure 2. The MnP or laccase activities of different strains varied greatly, and none of these isolates could secrete MnP and laccase at the same time. The strain CGMCC5.2 of *Agaricus bisporus* had neither MnP activity nor laccase activity, indicating that the fungus had no ability to secrete MnP or laccase. The white rote fungi *Hypsizygus marmoreus* (including strains GC-Hm06, GC-Hm23 and GC-Hm59) and *Lentinula edodes* GC-Le95 had no ability to secrete MnP, but the laccase secreted by these strains were active, among which GC-Le95 had the highest laccase activity of 1.82 U/mL. The edible fungi *Agrocybe cylindracea* strain GC-Ac2 and *Cyclocybe cylindracea* strain GC-Cc74 had strong ability to secrete MnP, and the MnP activity was greater than 1.50 U/mL, among which the MnP activity of *Agrocybe cylindracea* GC-Ac2 culture supernatant was as high as 2.37 U/mL. The purification process of MnP can be complex and time-consuming, resulting in relatively low yields (Zhang et al., 2018; Pech-Canul et al., 2020). Therefore, microbial strains with high MnP yield are valuable but currently scarce. This study shows for the first time that the specie *Agrocybe cylindracea* was able to produce MnP, emphasizing its novelty and potential importance in mycotoxin degradation. To further support this finding, we performed a search for edible fungi producing MnP in the Unified Protein Database.^2^ As shown in Supplementary Table S3, among the 14 identified species of edible fungi, *Agrocybe cylindracea* was not included. This indicates that the ability of *Agrocybe cylindracea* to produce MnP is a novel and unique characteristic.

Edible fungi are one of the main sources of laccase (a multicopper oxidase that catalyzes lignin degradation or polymerization) and MnP (a Mn^2+^-dependent lignin-degrading peroxidase) (Kumar and Arora, 2022; Zhang et al., 2022), the enzymes are shown to be catalytically active for mycotoxin bioremediation (Dellafiora et al., 2017; Loi et al., 2018; Xia et al., 2022; Lou et al., 2023). Our findings align with previous research that highlighted the key role of laccase and MnP in the degradation of mycotoxins. For instance, *Agaricus bisporus* CGMCC5.2 did not produce MnP or laccase, and its mycotoxin removal rate was low. In contrast, the other seven strains producing MnP or laccase displayed higher removal rates (over 70%).

### Pattern analysis of AFB_1_ and ZEN removal by *Agrocybe cylindracea* GC-Ac2

According to the above results, it can be seen that the strain *Agrocybe cylindracea* GC-Ac2 had the best removal effect on AFB_1_ and ZEN, had strong resistance to toxicity, and could secret high levels of MnP. The strain *Lentinula edodes* SD-Le95 performed best in AFB_1_ and ZEN removal and had high laccase activity, which deserves further study. Notebly, MnP has a higher redox potential compared to laccase, which enables it to oxidize more complex organic compounds, including polyphenols, methylated compounds, and nitrated aromatic compounds (Kumar and Arora, 2022). This property of MnP makes it particularly effective in detoxifying various mycotoxins found in feed and food, such as AFs, ZEN, DON, and FBs (Wang et al., 2019). Therefore, the strain *Agrocybe cylindracea* GC-Ac2 was primarily explored in this study.

As shown in Table 3, the removal rate of AFB_1_ by the culture supernatant (95.4%) of *Agrocybe cylindracea* GC-Ac2 was significantly higher than that of either mycelial adsorption (9.9%) or intracellular component (15.1%), indicating that the AFB_1_ removal was mainly performed by extracellular components in the culture supernatant. In addition, strain GC-Ac2 degraded 90.8% of ZEN through the culture supernatant, which was higher than the ZEN degraded by intracellular component (7.7%) and adsorbed by mycelia (36.8%), suggesting that the cell-free supernatant of the strain GC-Ac2 degraded ZEN effectively, and the adsorption of ZEN by active cells were also effective. Furthermore, as shown in Table 3, after the culture of strain GC-Ac2 for 10 days, the MnP activities in the culture supernatant and intracellular components were 2.01 U/mL and 0.21 U/mL, respectively. The results suggest that a small amount of MnPs are present in intracellular components. Based on previous reports, MnPs are extracellular enzymes commonly secreted by white and brown rot fungi (Chowdhary et al., 2019; Cagide and Castro-Sowinski, 2020; Obinger, 2022). Therefore, it is speculated that the MnPs in intracellular components are more likely to be the same as the MnPs in the culture supernatant, as these MnPs might be in a free state in the cytoplasm during the process of secretion into the culture supernatant. Additionally, fresh mycelial pellets were used to detect intracellular MnPs activity, and the metabolism producing MnPs in fresh mycelial pellets might be still active, so that it was possible to detect extracellular MnPs in intracellular components.

**Table 3:** Differences in AFB_1_ and ZEN removal abilities among different cell components.

Component	AFB_1_ residue ± SD (ng/mL)		AFB_1_ removal (%)	ZEN residue ± SD (ng/mL)		ZEN removal (%)	MnP activity (U/mL)
Control	484.2 ± 3.8	*A		472.7 ± 2.0	*A		
Mycelial adsorption	429.8 ± 4.6	C	9.9	298.7 ± 3.0	C	36.8	
Intracellular component	405.6 ± 5.0	B	15.1	436.2 ± 2.2	B	7.7	0.21 ± 0.008
Culture supernatant	21.9 ± 0.3	D	95.4	43.5 ± 1.7	D	90.8	2.01 ± 0.091

*AFB_1_ and ZEN residues followed by different capital letters are statistically different according to Tukey–Kramer Multiple Comparison test (*p* < 0.001).

### Optimal conditions for simultaneous degradation of AFB_1_ and ZEN in the culture supernatant of *Agrocybe cylindracea* GC-Ac2

**Figure 3:**
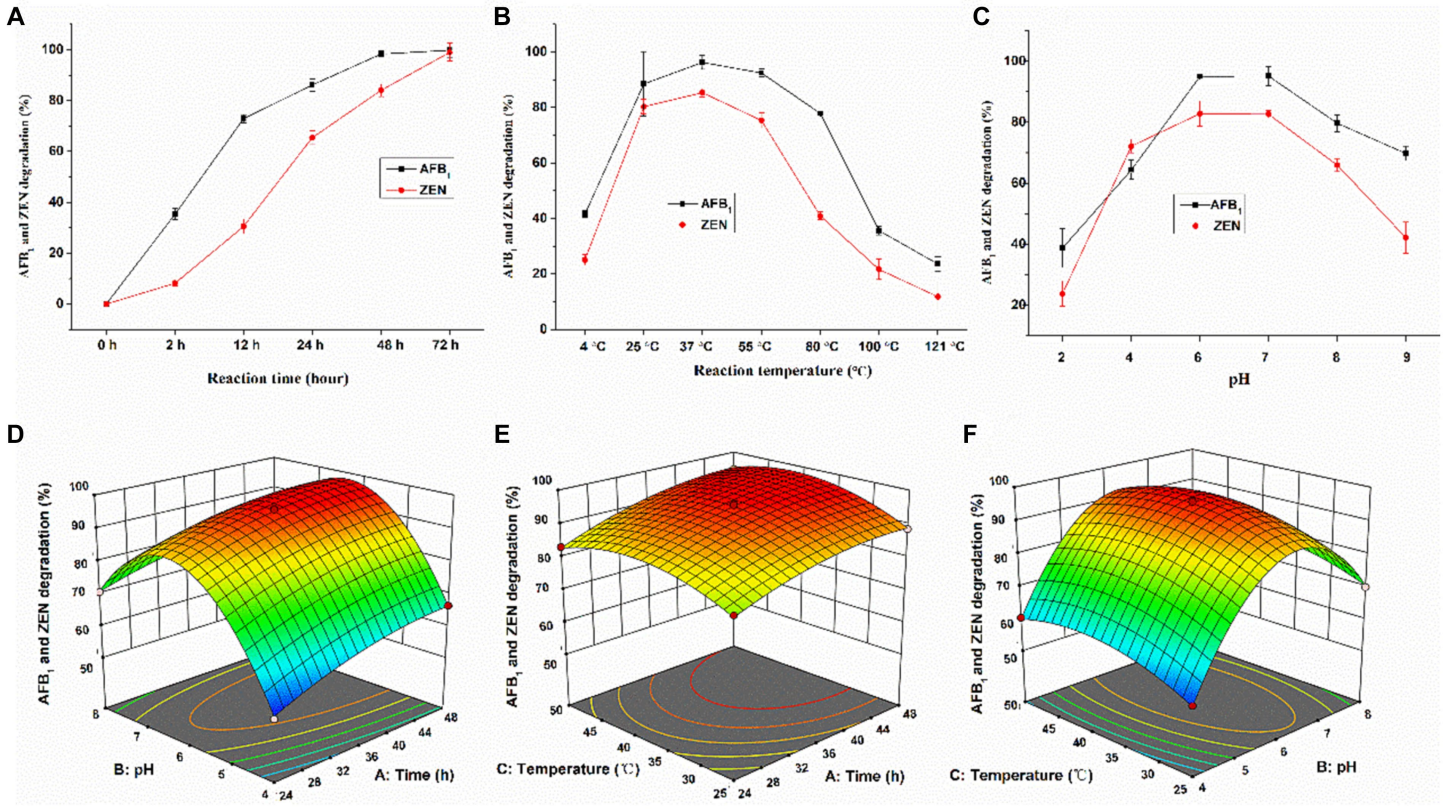
Optimal conditions for culture supernatant of *Agrocybe cylindracea* GC-Ac2 to simultaneously degrade AFB_1_ and ZEN. Effects of **(A)** a ranged reaction time when the pH value was 5.8 and reaction temperature was 37°C, **(B)** different reaction temperature when the reaction time was 48 h and the pH value was 5.8, and **(C)** different pH values when reaction time was 48 h and reaction temperature was 37°C on the silmultaneous degradation of 100 ng/mL AFB_1_ and 200 ng/mL ZEN by *Agrocybe cylindracea* GC-Ac2 culture supernatant. Response plots between two parameters for the simultaneous degradation rate of AFB_1_ and ZEN: **(D)** reaction time and pH; **(E)** reaction time and reaction temperature; and **(F)** pH and reaction temperature.

The time course of degradation of AFB_1_ and ZEN by GC-Ac2 culture supernatant were shown in Figure 3A. The degradation rate of AFB_1_ was 35.3% at 2 h, and gradually ascended to 72.9 and 86.1% at 12 h and 24 h, respectively. Approximately 100% degradation of AFB_1_ occurred after 48 h of reaction. In comparison, ZEN initially degraded slowly, with degradation percentages of 8.2 and 30.5% at 2 h and 12 h, respectively, but the percentages steadily increased and then almost 100% ZEN was degraded within 72 h of reaction. As shown in Figure 3B, the degradation of AFB_1_ and ZEN by GC-Ac2 culture supernatant was temperature-sensitive within the range of 4–121°C. The maximum degradation occurred at around 37°C, and then the degradation rates decreased gradually with the increase of the temperature. As shown in Figure 3C, the optimal pH value of the culture supernatant for degrading AFB_1_ and ZEN was between 6 and 7. When the pH was below 2.0 or above 9.0, the culture supernatant was completely inactivated.

The analysis of variance of the quadratic model is shown in Supplementary Table S4, which intuitively reflects the influence of various factors on the response value. According to the detailed results, the regression equation is: Y = 95.20 + 5.41A + 7.64B + 2.40C – 1.36AB + 0.70 AC – 0.69 BC – 3.00A^2^–25.02B^2^–5.66C^2^, where A, B, C, AB, A^2^, B^2^ and C^2^ are important model terms. The response surface and contour plots are shown in Figures 3D–F. According to the Design-Expert 12.0.3 software, the optimal culture conditions were pH 6, temperature 37.4°C and culture time 37.9 h. Under optimal culture conditions, the simultaneous degradation rate of AFB_1_ and ZEN was 96.0%.

### Toxicity analysis of AFB_1_ and ZEN degradation products in the culture supernatant of *Agrocybe cylindracea* GC-Ac2

The structures of AFB_1_ and ZEN have similar aromatic structures. MnP can generate free radicals to oxidize the aromatic structure, thereby breaking the covalent bond and generating enzymatic degradation products of AFB_1_-diol and 15-OH-ZEN, whose toxicity is reduced greatly compared with AFB_1_ and ZEN (Wang et al., 2011; Qin et al., 2021). However, it is important to note that not all mycotoxin degradation products are necessarily less toxic or non-toxic. Take the MnP enzyme in the white rot fungus *Irpex lacteus* CD2 as an example. It can oxidize AFB_1_ to AFB_1_-8,9-epoxide, which is actually more toxic than AFB_1_ itself (Wang et al., 2019). In this study, the presence of AFB_1_-8,9-epoxide was not detected in the culture supernatant of *Agrocybe cylindracea* GC-Ac2.

**Figure 4:**
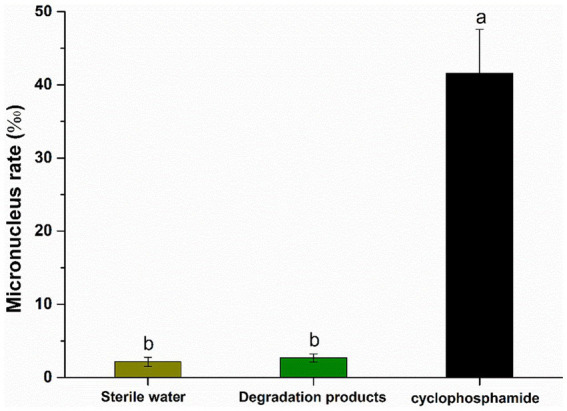
Results of micronucleus test of polychromatic erythrocytes in the bone marrow of mice. Bars with different letters are significantly different for *p* < 0.001 (Tukey–Kramer multiple comparison test).

As shown in Figure 4, there was no significant difference (*p* > 0.05) in the bone marrow erythrocytes of the experimental mouse (intragastrically fed with degradation products) and the negative control (intragastrically fed with sterile water). However, the micronucleus rate in the positive control group (intragastrically fed with cyclophosphamide) was 41.6‰, which was significantly different from the experimental mouse group (*p* < 0.001). This indicates that the degradation products of AFB_1_ and ZEN did not increase the micronucleus rate in mouse bone marrow erythrocytes. These results suggested that AFB_1_ and ZEN degraded products are non-toxic. It certainly need further study to figure out the degradation pathways and the molecular structures of the degradation products. However, if the residual toxicity of degradation products is properly carefully monitored, *Agrocybe cylindracea* GC-Ac2 or its culture supernatant could be used as candidate biomaterials for the simultaneous degradation of multiple mycotoxins in food and feed.

### MnP activity dynamics and AFB_1_/ZEN removal dynamics in the culture supernatant of *Agrocybe cylindracea* GC-Ac2

**Figure 5:**
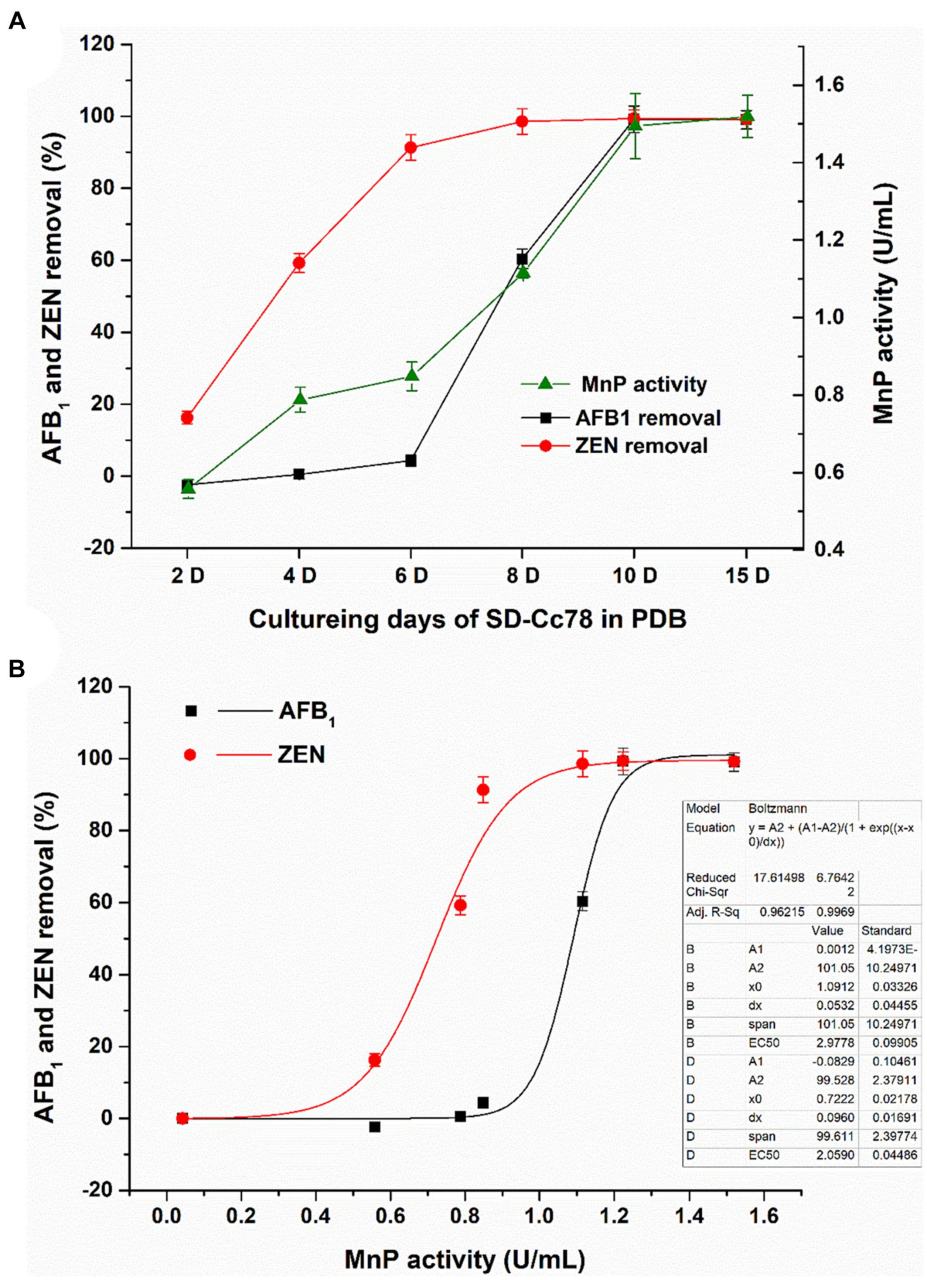
**(A)** Dynamics of MnP activity in the culture supernatant of and dynamics of AFB_1_/ZEN removal by *Agrocybe cylindracea* GC-Ac2, with the increase of co-culturing time. **(B)** Mathematical correlation of MnP activity with AFB_1_/ZEN removal obtained using OriginPro9.0 and Boltzmann equation model.

As can be seen from Figure 5A, the MnP activity of the GC-Ac2 culture supernatant gradually increased with the increase of culture time and became stable on the 10th day. The removal rate of AFB_1_ increased gradually and reached 100% on the 10th day, which was basically consistent with the variation trend of MnP activity. However, the removal rate of ZEN increased rapidly from the second day, reached 91% on the sixth day, and then became stable. The removal rate of ZEN was not completely consistent with the variation trend of MnP activity. It can be explained that the removal of ZEN may be the result of absorption in the early growth stage plus the biodegradation, because the adsorption of ZEN on GC-Ac2 mycelia could not be ignored (Table 3). However, as shown in Figure 5B, different trends did not necessarily mean they were uncorrelated. The Boltzmann equation model is a useful tool for studying the dynamics of microorganism growth, enzyme production, metabolism, etc. Using the Boltzmann equation model, MnP activities on the ordinate were plotted against AFB_1_ and ZEN removal rates on the abscissa. The correlation of MnP activity with AFB_1_ removal and ZEN removal resulted in two S-sharped curve equations with R_1_^2^ = 0.96 and R_2_^2^ = 0.99, respectively, indicating that AFB_1_ and ZEN removal varied with changes in MnP activity. Even so, we could not conclude that the removal was the function of MnP. This might be due to: (1) other active components such as ligninolytic enzymes, which had similar dynamics of MnP activity and played a role in the removal (Peng et al., 2018); and (2) MnP interacted with other active components to play a removal role together (Wang and Xie, 2020).

### Effects of heat, proteinase K + SDS, EDTA, NaN_3_, Mn^2+^ (10 mM), H_2_O_2_ (5 mM) and Mn^2+^ (1 mM) + H_2_O_2_ (0.1 mM) + malonate (50 mM) on the activity of the culture supernatant to degrade AFB_1_ and ZEN

**Figure 6:**
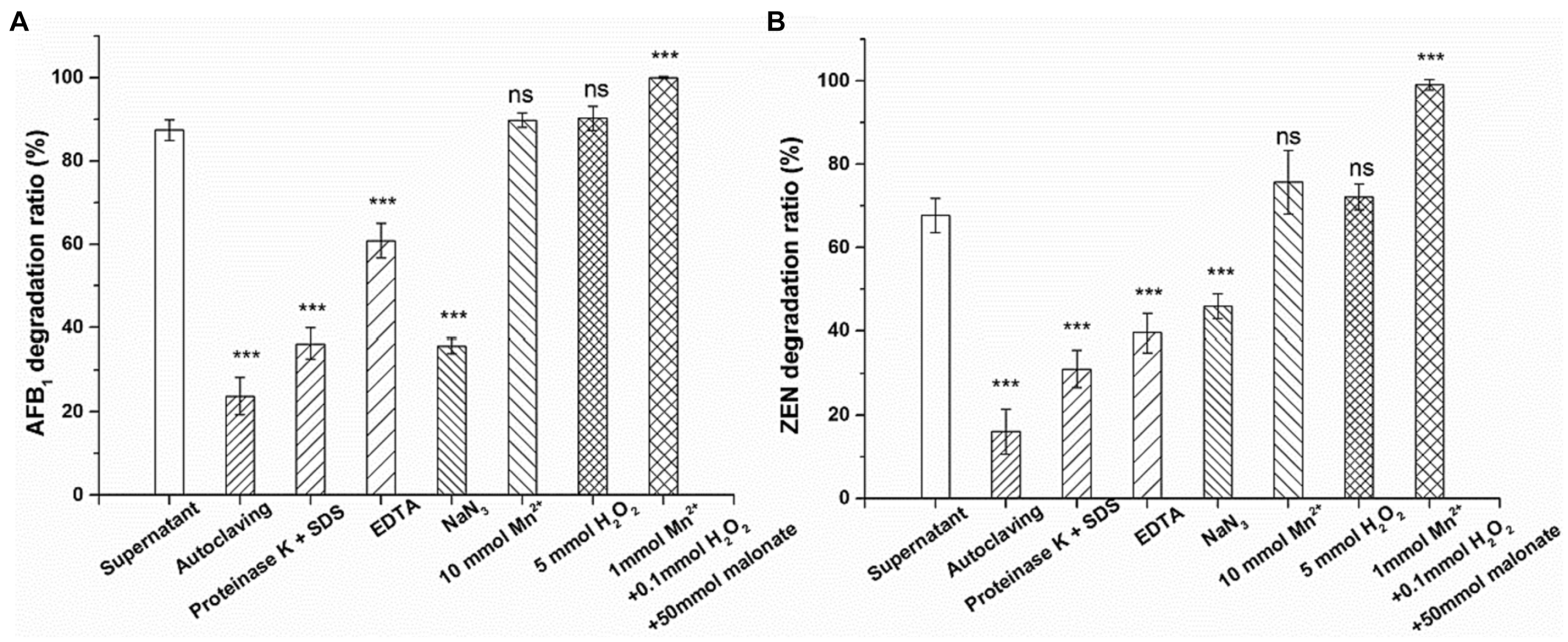
Effects of autoclaving, proteinase K + SDS, EDTA, NaN_3_, Mn^2+^ (10 mM), H_2_O_2_ (5 mM) and Mn^2+^ (1 mM) + H_2_O_2_ (0.1 mM) + malonate (50 mM) on the activity of the culture supernatant of *Agrocybe cylindracea* GC-Ac2 to degrade **(A)** AFB_1_ and **(B)** ZEN. Asterisks indicate statistically significant values at *p* > 0.05 (ns), *p* < 0.05 (*), *p* < 0.01 (**) or = *p* < 0.001 (***) by one-way ANOVA.

To further investigate the active components that degrade AFB_1_ and ZEN in the culture supernatant of *Agrocybe cylindracea* GC-Ac2, inactivation or activation treatments were conducted. Generally, enzymes could be inactivated by treatments with heat and proteinase K plus SDS (Wang et al., 2017). As a metal chelating agent, EDTA at 1 mM could inhibit the peroxidase activity completely (Kanayama et al., 2002). Sodium azide (NaN_3_), as an inhibitor, inhibits the activity of peroxidase by binding to iron ions (Gao et al., 2018). As shown in Figure 6, after incubation at 37°C for 24 h, the culture supernatant of GC-Ac2 degraded approximately 87.4% of AFB_1_ and 67.7% of ZEN. However, after a treatment with autoclaving at 121°C for 30 min, the degradation rates of AFB_1_ and ZEN dropped significantly to 23.8 and 16.1%, respectively; after a treatment with proteinase K plus SDS, the degradation rates of AFB1 and ZEN dropped significantly to 36.3 and 31.1%, respectively, indicating that the active components are thermo-sensitive enzymes. After treatment with EDTA (10 mM) and NaN3, the degradation rates of AFB_1_ and ZEN were significantly reduced, indicating that the active components contain peroxidase.

MnP is a heme-containing peroxidase commonly secreted by edible fungi that requires H_2_O_2_ as an oxidant to function. MnP catalysis relies on manganese, with Mn^2+^ serving as the preferred substrate for oxidization to Mn^3+^. Mn^3+^ is unstable and can chelate with carboxylic acids such as malonate and oxalic acid to form a high REDOX potential, thereby oxidizing various phenolic substrates, such as simple phenols, lignin and toxins (Chowdhary et al., 2019). Xia et al. (2022) and Yehia (2014) reported that the degradation of AFB_1_ by MnP requires the participation of Mn^2+^ and H_2_O_2_, but a high concentration of MnSO_4_ (1 mM) and H_2_O_2_ (10 mM) inhibited AFB_1_ degradation. In contrast to earlier findings, however, the results showed that 10 mM H_2_O_2_ or 5 mM Mn^2+^ had no significant effect on the degradation rate (Figure 6). In addition, without adding any H_2_O_2_ or Mn^2+^, the culture supernatant of *Agrocybe cylindracea* GC-Ac2 still has high mycotoxin degradation activity. The account assumes that although the culture supernatant of *Agrocybe cylindracea* GC-Ac2 is rich in MnP, its potential on mycotoxins degradation has not yet been realized. White rot fungi are a physiological group that produces synergistic ligninolytic enzymes and have excellent capacities to degrade lignin and lignin-like substances (Cagide and Castro-Sowinski, 2020). Therefore, other ligninolytic enzymes may act directly on AFB_1_ and ZEN degradation without the assistance of H_2_O_2_ or Mn^2+^.

Wang et al. (2019) reported that MnP degraded AFB_1_ and ZEN only in the presence of a dicarboxylic acid, such as malonate. Some studies also reported that MnP has the potential to degrade dyes, phenol and toxins in the system containing H_2_O_2_, Mn^2+^ and dicarboxilic acids, such as malonate (Xu et al., 2017; Zhang et al., 2018, 2020). In our experiments, Mn^2+^ (1 mM), H_2_O_2_ (0.1 mM) and malonate (50 mM) were used to activate the catalytic function of MnP. Under these conditions, AFB_1_ and ZEN in the culture supernatant of *Agrocybe cylindracea* GC-Ac2 were completely degraded (Figure 6). This suggested that Mn^2+^, H_2_O_2_ and malonic acid activated the previously quiescent MnP activity in the culture supernatant of *Agrocybe cylindracea* GC-Ac2.

These results indicate that the active components of mycotoxin degradation in the culture supernatant of *Agrocybe cylindracea* GC-Ac2 was a complex enzyme system. The enzyme system may include MnP and other ligninolytic enzymes. The characteristics of this enzyme system include: (1) thermo-sensitivity; (2) high activity and broad substrate specificity, able to act on many structurally different mycotoxins; and (3) Mn^2+^, H_2_O_2_ and malonate enhancing the effects on mycotoxins by stimulating the activity of MnP.

### Cultivation of *Agrocybe cylindracea* GC-Ac2 in a mycotoxins-contaminated mushroom substrate

As shown in Table 4, the substrates were contaminated with eleven mycotoxins of AFB_1_, ZEN, 15A-DON, 3A-DON, DON, FB_1_, FB_2_, FB_3_, T-2, OTA and ST. Surprisingly, after culturing the strain GC-Ac2 in the mushroom substrate for 20 days, the strain *Agrocybe cylindracea* GC-Ac2 could degrade nine mycotoxins including AFB_1_, ZEN, 15A-DON, FB_1_, FB_2_, FB_3_, T-2, OTA and ST with different degradation rates. GC-Ac2 could remove more than 70% of AFB_1_, ZEN, FB_3_, T_2_ and ST, more than 20% of 15A-DON, FB_1_, FB_2_ and OTA, but could not remove DON and 3A-DON. White rot fungi possess unique oxidative and extracellular ligninolytic systems with low substrate specificity, enabling them to transform or degrade different environmental contaminants (Zhuo and Fan, 2021). In the previous section, *Agrocybe cylindracea* GC-Ac2 was considered to have a complex enzyme system. It might be this complex enzyme system that enables *Agrocybe cylindracea* GC-Ac2 to degrade a variety of mycotoxins with different structures.

**Table 4:** Degradation of mycotoxins by strain *Agrocybe cylindracea* GC-Ac2 grown in a mushroom substrate containing naturally contaminated maize, wheat and peanut meal, after 20 days of culture.

Mycotoxins	Control	GC-Ac2	Removal (%)
AFB_1_	135.6 ± 2.9	3.1 ± 1.0	97.7
ZEN	7.4 ± 2.5	1.3 ± 0.2	81.8
15A-DON	23.1 ± 0.3	10.2 ± 5.6	56.0
3A-DON	3.2 ± 0.4	5.4 ± 1.2	0.0
DON	32.4 ± 0.1	38.1 ± 34.1	0.0
FB_1_	282.7 ± 4.4	224.8 ± 13.1	20.5
FB_2_	77.9 ± 4.8	45.7 ± 6.7	41.3
FB_3_	48.6 ± 2.9	14.1 ± 2.4	70.9
T-2	80.4 ± 6.8	7.2 ± 0.9	91.0
OTA	169.9 ± 6.6	122.6 ± 6.2	27.8
ST	78.9 ± 5.7	0.6 ± 0.3	99.2
AFB_1_-8,9-epoxide	Not detected	Not detected	

## Conclusion

Through the screening of strain *Agrocybe cylindracea* GC-Ac2, its degradation efficiency against multiple mycotoxins, including AFB_1_, ZEN, 15A-DON, FB_1_, FB_2_, FB_3_, T-2, OTA, and ST, was revealed. Additionally, this study has demonstrated, for the first time, that *Agrocybe cylindracea* can produce highly active MnP enzyme. The mechanism of degradation of AFB_1_ and ZEN by the culture supernatant of *Agrocybe cylindracea* GC-Ac2 was determined to be catalyzed by a complex enzyme system, which might include MnP and other ligninolytic enzymes. However, further research is needed to gain a deeper understanding of this enzyme system in the future. To fully realize the potential of strain *Agrocybe cylindracea* GC-Ac2, further research is needed to explore the degradation pathways and products of mycotoxins. Furthermore, although this study focused primarily on AFB_1_ and ZEN, the characterization and mechanism by which strain *Agrocybe cylindracea* GC-Ac2 simultaneously degrades multiple mycotoxins certainly deserve further attention.

## Data availability statement

The original contributions presented in the study are included in the article/Supplementary material, further inquiries can be directed to the corresponding author.

## Author contributions

CG: Methodology, Writing – original draft, Formal analysis, Investigation. LF: Investigation, Methodology, Writing – original draft. QY: Methodology, Writing – review & editing. MN: Methodology, Writing – original draft. BZ: Methodology, Writing – original draft. XR: Methodology, Writing – original draft, Writing – review & editing.

## Footnotes

^1^ https://cgmcc.net

^2^ www.uniprot.org

## References

[ref1] Adebo O. A. Njobeh P. B. Gbashi S. Nwinyi O. C. Mavumengwana V. (2017). Review on microbial degradation of aflatoxins. Crit. Rev. Food Sci. Nutr. 57, 3208–3217. doi: 10.1080/10408398.2015.1106440 26517507

[ref2] Adebo O. A. Njobeh P. B. Mavumengwana V. (2016). Degradation and detoxification of AFB_1_ by *Staphylocococcus warneri*, *Sporosarcina sp*. and *Lysinibacillus fusiformis*. Food Control 68, 92–96. doi: 10.1016/j.foodcont.2016.03.021

[ref3] Bi K. Zhang W. Xiao Z. Zhang D. (2018). Characterization, expression and application of a zearalenone degrading enzyme from *Neurospora crassa*. AMB Express 8:194. doi: 10.1186/s13568-018-0723-z, PMID: 30570697 PMC6301899

[ref4] Cagide C. Castro-Sowinski S. (2020). Technological and biochemical features of lignin-degrading enzymes: a brief review. Environ. Sustain. 3, 371–389. doi: 10.1007/s42398-020-00140-y

[ref5] Chowdhary P. Shukla G. Raj G. Ferreiraz L. F. R. Bharagava R. N. (2019). Microbial manganese peroxidase: a ligninolytic enzyme and its ample opportunities in research. SN Appl. Sci. 1, 1–12. doi: 10.1007/s42452-018-0046-3

[ref6] Dellafiora L. Galaverna G. Reverberi M. Dall’Asta C. (2017). Degradation of aflatoxins by means of laccases from *Trametes versicolor*: an in silico insight. Toxins (Basel) 9:17. doi: 10.3390/toxins9010017, PMID: 28045427 PMC5308249

[ref7] Escriva L. Font G. Manyes L. Berrada H. (2017). Studies on the presence of mycotoxins in biological samples: an overview. Toxins (Basel) 9:251. doi: 10.3390/toxins9080251, PMID: 28820481 PMC5577585

[ref8] Fanelli F. Geisen R. Schmidt-Heydt M. Logrieco A. F. Mulè G. (2016). Light regulation of mycotoxin biosynthesis: new perspectives for food safety. World Mycotoxin J. 9, 129–146. doi: 10.3920/wmj2014.1860

[ref9] Gao C. Chang P. Yang L. Wang Y. Zhu S. Shan H.. (2018). Neuroprotective effects of hydrogen sulfide on sodium azide-induced oxidative stress in PC12 cells. Int. J. Mol. Med. 41, 242–250. doi: 10.3892/ijmm.2017.3227, PMID: 29115393 PMC5746291

[ref10] Guan Y. Chen J. Nepovimova E. Long M. Wu W. Kuca K. (2021). Aflatoxin detoxification using microorganisms and enzymes. Toxins (Basel) 13:46. doi: 10.3390/toxins13010046, PMID: 33435382 PMC7827145

[ref11] Guo Y. Qin X. Tang Y. Ma Q. Zhang J. Zhao L. (2020). CotA laccase, a novel aflatoxin oxidase from *Bacillus licheniformis*, transforms aflatoxin B_1_ to aflatoxin Q_1_ and epi-aflatoxin Q_1_. Food Chem. 325:126877. doi: 10.1016/j.foodchem.2020.126877, PMID: 32387986

[ref12] Haidukowski M. Casamassima E. Cimmarusti M. T. Branà M. T. Longobardi F. Acquafredda P.. (2019). Aflatoxin B_1_-adsorbing capability of *Pleurotus eryngii* mycelium: efficiency and modeling of the process. Front. Microbiol. 10:1386. doi: 10.3389/fmicb.2019.01386, PMID: 31293538 PMC6604724

[ref13] Haque M. A. Wang Y. Shen Z. Li X. Saleemi M. K. He C. (2020). Mycotoxin contamination and control strategy in human, domestic animal and poultry: a review. Microb. Pathog. 142:104095. doi: 10.1016/j.micpath.2020.104095, PMID: 32097745

[ref14] Kanayama N. Suzuki T. Kawai K. (2002). Purification and characterization of an alkaline manganese peroxidase from *aspergillus terreus* LD-1. J. Biosci. Bioeng. 93, 405–410. doi: 10.1016/s1389-1723(02)80075-5, PMID: 16233222

[ref15] Kumar A. Arora P. K. (2022). Biotechnological applications of manganese peroxidases for sustainable management. Front. Environ. Sci. 10:365. doi: 10.3389/fenvs.2022.875157

[ref16] Kunze G. Branà M. T. Cimmarusti M. T. Haidukowski M. Logrieco A. F. Altomare C. (2017). Bioremediation of aflatoxin B_1_-contaminated maize by king oyster mushroom (*Pleurotus eryngii*). PLoS One 12:e0182574. doi: 10.1371/journal.pone.0182574, PMID: 28771640 PMC5542706

[ref17] Li J. Huang J. Jin Y. Wu C. Shen D. Zhang S.. (2018). Mechanism and kinetics of degrading aflatoxin B_1_ by salt tolerant *Candida versatilis* CGMCC 3790. J. Hazard. Mater. 359, 382–387. doi: 10.1016/j.jhazmat.2018.05.053, PMID: 30053743

[ref18] Loi M. Fanelli F. Cimmarusti M. T. Mirabelli V. Haidukowski M. Logrieco A. F.. (2018). In vitro single and combined mycotoxins degradation by Ery4 laccase from *Pleurotus eryngii* and redox mediators. Food Control 90, 401–406. doi: 10.1016/j.foodcont.2018.02.032

[ref19] Loi M. Fanelli F. Liuzzi V. C. Logrieco A. F. Mule G. (2017). Mycotoxin biotransformation by native and commercial enzymes: present and future perspectives. Toxins (Basel) 9:111. doi: 10.3390/toxins9040111 28338601 PMC5408185

[ref20] Loi M. Renaud J. B. Rosini E. Pollegioni L. Vignali E. Haidukowski M.. (2020). Enzymatic transformation of aflatoxin B_1_ by Rh-DypB peroxidase and characterization of the reaction products. Chemosphere 250:126296. doi: 10.1016/j.chemosphere.2020.126296, PMID: 32135437

[ref21] Lou H. Yang C. Gong Y. Li Y. Li Y. Tian S.. (2023). Edible fungi efficiently degrade aflatoxin B_1_ in cereals and improve their nutritional composition by solid-state fermentation. J. Hazard. Mater. 451:131139. doi: 10.1016/j.jhazmat.2023.131139 36921416

[ref22] Luo Y. Liu X. Li J. (2018). Updating techniques on controlling mycotoxins: a review. Food Control 89, 123–132. doi: 10.1016/j.foodcont.2018.01.016

[ref23] Luo X. Wang R. Wang L. Wang Y. Chen Z. (2013). Structure elucidation and toxicity analyses of the degradation products of aflatoxin B_1_ by aqueous ozone. Food Control 31, 331–336. doi: 10.1016/j.foodcont.2012.10.030

[ref24] Mitchell N. J. Bowers E. Hurburgh C. Wu F. (2016). Potential economic losses to the US corn industry from aflatoxin contamination. Food Addit. Contam. Part A 33, 540–550. doi: 10.1080/19440049.2016.1138545, PMID: 26807606 PMC4815912

[ref25] Obinger C. (2022). On “Purification and characterization of an extracellular Mn(II)-dependent peroxidase from the lignin-degrading basidiomycete, Phanerochaete chrysosporium” by Jeffrey K. Glenn and Michael H. Gold. Arch. Biochem. Biophys. 726:109257. doi: 10.1016/j.abb.2022.109257 35452624

[ref26] Pankaj S. K. Shi H. Keener K. M. (2018). A review of novel physical and chemical decontamination technologies for aflatoxin in food. Trends Food Sci. Technol. 71, 73–83. doi: 10.1016/j.tifs.2017.11.007

[ref27] Pech-Canul A. C. Carrillo-Campos J. Ballinas-Casarrubias M. L. Solis-Oviedo R. L. Hernandez-Rascon S. K. Hernandez-Ochoa L. R.. (2020). Functional expression and one-step protein purification of manganese peroxidase 1 (rMnP1) from *Phanerochaete chrysosporium* using the *E. coli*-expression system. Int. J. Mol. Sci. 21:416. doi: 10.3390/ijms21020416, PMID: 31936493 PMC7013543

[ref28] Peng Z. Chen L. Zhu Y. Huang Y. Hu X. Wu Q.. (2018). Current major degradation methods for aflatoxins: a review. Trends Food Sci. Technol. 80, 155–166. doi: 10.1016/j.tifs.2018.08.009

[ref29] Qin X. Su X. Tu T. Zhang J. Wang X. Wang Y.. (2021). Enzymatic degradation of multiple major mycotoxins by dye-decolorizing peroxidase from *Bacillus subtilis*. Toxins (Basel) 13:429. doi: 10.3390/toxins13060429, PMID: 34205294 PMC8235724

[ref30] Schatzmayr G. Streit E. (2013). Global occurrence of mycotoxins in the food and feed chain: facts and figures. World Mycotoxin J. 6, 213–222. doi: 10.3920/wmj2013.1572

[ref31] Shi H. Cooper B. Stroshine R. L. Ileleji K. E. Keener K. M. (2017). Structures of degradation products and degradation pathways of aflatoxin B_1_ by high-voltage atmospheric cold plasma (HVACP) treatment. J. Agric. Food Chem. 65, 6222–6230. doi: 10.1021/acs.jafc.7b01604 28643515

[ref32] Terzi V. Tumino G. Stanca A. M. Morcia C. (2014). Reducing the incidence of cereal head infection and mycotoxins in small grain cereal species. J. Cereal Sci. 59, 284–293. doi: 10.1016/j.jcs.2013.10.005

[ref33] Tolosa J. Rodríguez-Carrasco Y. Ruiz M. J. Vila-Donat P. (2021). Multi-mycotoxin occurrence in feed, metabolism and carry-over to animal-derived food products: a review. Food Chem. Toxicol. 158:112661. doi: 10.1016/j.fct.2021.112661, PMID: 34762978

[ref34] Wang J. Ogata M. Hirai H. Kawagishi H. (2011). Detoxification of aflatoxin B_1_ by manganese peroxidase from the white-rot fungus *Phanerochaete sordida* YK-624. FEMS Microbiol. Lett. 314, 164–169. doi: 10.1111/j.1574-6968.2010.02158.x, PMID: 21118293

[ref35] Wang X. Qin X. Hao Z. Luo H. Yao B. Su X. (2019). Degradation of four major mycotoxins by eight manganese peroxidases in presence of a dicarboxylic acid. Toxins (Basel) 11:566. doi: 10.3390/toxins11100566, PMID: 31569657 PMC6833064

[ref36] Wang J. Xie Y. (2020). Review on microbial degradation of zearalenone and aflatoxins. Grain Oil Sci. Technol. 3, 117–125. doi: 10.1016/j.gaost.2020.05.002

[ref37] Wang Y. Zhao C. Zhang D. Zhao M. Zheng D. Lyu Y.. (2017). Effective degradation of aflatoxin B_1_ using a novel thermophilic microbial consortium TADC7. Bioresour. Technol. 224, 166–173. doi: 10.1016/j.biortech.2016.11.033, PMID: 27866802

[ref38] Wild C. P. Gong Y. Y. (2010). Mycotoxins and human disease: a largely ignored global health issue. Carcinogenesis 31, 71–82. doi: 10.1093/carcin/bgp264, PMID: 19875698 PMC2802673

[ref39] Wu Y. Cheng J. H. Sun D. W. (2021). Blocking and degradation of aflatoxins by cold plasma treatments: applications and mechanisms. Trends Food Sci. Technol. 109, 647–661. doi: 10.1016/j.tifs.2021.01.053

[ref40] Xia Y. He R. Sun Y. Zhou H. Gao M. Hu X.. (2022). Food-grade expression of manganese peroxidases in *recombinant Kluyveromyces* lactis and degradation of aflatoxin B_1_ using fermentation supernatants. Front. Microbiol. 12:821230. doi: 10.3389/fmicb.2021.821230, PMID: 35237243 PMC8882868

[ref41] Xu H. Guo M. Y. Gao Y. H. Bai X. H. Zhou X. W. (2017). Expression and characteristics of manganese peroxidase from *Ganoderma lucidum* in Pichia pastoris and its application in the degradation of four dyes and phenol. BMC Biotechnol. 17:19. doi: 10.1186/s12896-017-0338-5, PMID: 28231778 PMC5324234

[ref42] Yehia R. S. (2014). Aflatoxin detoxification by manganese peroxidase purified from *Pleurotus ostreatus*. Braz. J. Microbiol. 45, 127–134. doi: 10.1590/s1517-83822014005000026, PMID: 24948923 PMC4059287

[ref43] Yue X. Ren X. Fu J. Wei N. Altomare C. Haidukowski M.. (2022). Characterization and mechanism of aflatoxin degradation by a novel strain of *Trichoderma reesei* CGMCC3.5218. Front. Microbiol. 13:1003039. doi: 10.3389/fmicb.2022.1003039, PMID: 36312918 PMC9611206

[ref44] Zain M. E. (2011). Impact of mycotoxins on humans and animals. J. Saudi Chem. Soc. 15, 129–144. doi: 10.1016/j.jscs.2010.06.006

[ref45] Zhang S. Dong Z. Shi J. Yang C. Fang Y. Chen G.. (2022). Enzymatic hydrolysis of corn Stover lignin by laccase, lignin peroxidase, and manganese peroxidase. Bioresour. Technol. 361:127699. doi: 10.1016/j.biortech.2022.127699, PMID: 35905874

[ref46] Zhang H. Zhang X. Geng A. (2020). Expression of a novel manganese peroxidase from *Cerrena unicolor* BBP6 in *Pichia pastoris* and its application in dye decolorization and PAH degradation. Biochem. Eng. J. 153:107402. doi: 10.1016/j.bej.2019.107402

[ref47] Zhang H. Zhang J. Zhang X. Geng A. (2018). Purification and characterization of a novel manganese peroxidase from white-rot fungus *Cerrena unicolor* BBP6 and its application in dye decolorization and denim bleaching. Process Biochem. 66, 222–229. doi: 10.1016/j.procbio.2017.12.011

[ref48] Zhu Y. Hassan Y. I. Watts C. Zhou T. (2016). Innovative technologies for the mitigation of mycotoxins in animal feed and ingredients-a review of recent patents. Anim. Feed Sci. Technol. 216, 19–29. doi: 10.1016/j.anifeedsci.2016.03.030

[ref49] Zhuo R. Fan F. (2021). A comprehensive insight into the application of white rot fungi and their lignocellulolytic enzymes in the removal of organic pollutants. Sci. Total Environ. 778:146132. doi: 10.1016/j.scitotenv.2021.146132, PMID: 33714829

