# Supplementary material for: Characterization and mechanism of simultaneous degradation of aflatoxin B1 and zearalenone by an edible fungus of Agrocybe cylindracea GC-Ac2
Source: Front Microbiol. 2024 Feb 13;15:1292824. doi: 10.3389/fmicb.2024.1292824 (PMC10897045; doi:10.3389/fmicb.2024.1292824)
Supplement: Supplementary file 1 [file Table_1.docx]

**Table S1**. Factors and coded value of Box-Behnken design for the simultaneous degradation of AFB1 and ZEN by *C. cylindracea* SD-Cc78 culture supernatant.

| **Term** | **Variable** | **Coded level** | | |
| --- | --- | --- | --- | --- |
|  |  | **-1** | **0** | **1** |
| A | Rection time (hour) | 24 | 36 | 48 |
| B | Temperature (℃) | 25 | 37.5 | 50 |
| C | pH | 4 | 6 | 8 |

**Table S2.** Box-Behnken experimental design matrix and response values for the simultaneous degradation of AFB1 and ZEN by the *C. cylindracea* SD-Cc78 culture supernatant.

| **Run** | **Rection time (A)** | **Rection Temperature (B)** | **pH (C)** | **AFB_1_ degradation (Y)** |
| --- | --- | --- | --- | --- |
| 1 | 0 | 0 | 0 | 95.03 |
| 2 | 0 | -1 | -1 | 54.00 |
| 3 | 1 | 0 | -1 | 88.53 |
| 4 | 0 | 0 | 0 | 95.03 |
| 5 | -1 | 1 | 0 | 70.66 |
| 6 | 0 | 0 | 0 | 94.33 |
| 7 | 0 | 0 | 0 | 95.63 |
| 8 | 0 | 0 | 0 | 96.01 |
| 9 | 0 | -1 | 1 | 60.43 |
| 10 | -1 | -1 | 0 | 52.01 |
| 11 | 1 | 1 | 0 | 79.66 |
| 12 | -1 | 0 | -1 | 80.02 |
| 13 | 0 | 1 | -1 | 70.09 |
| 14 | 1 | -1 | 0 | 66.43 |
| 15 | -1 | 0 | 1 | 83.15 |
| 16 | 1 | 0 | 1 | 94.48 |
| 17 | 0 | 1 | 1 | 73.66 |

**Table S3. The edible fungi species producing MnP searched from Unified Protein Database** (www.uniprot.org)**.**

| **Number** | **Edible fungi** | **Species** | **UniProt ID** | **Length of the Mnp** |
| --- | --- | --- | --- | --- |
| 1 | White rot fungus  Or  Mushroom | [*Agaricus bisporus*](https://www.uniprot.org/taxonomy/5341) | Q5TJC2 | 354AA |
| 2 |  | [*Agrocybe praecox*](https://www.uniprot.org/taxonomy/71668) | [G4WG41](https://www.uniprot.org/uniprotkb/G4WG41/entry) | 358 AA |
| 3 |  | [*Ceriporiopsis subvermispora*](https://www.uniprot.org/taxonomy/42742) | [Q9UW38](https://www.uniprot.org/uniprotkb/Q9UW38/entry) | 389 AA |
| 4 |  | *Cerrena unicolor* | [A0A7D5FUQ6](https://www.uniprot.org/uniprotkb/A0A7D5FUQ6/entry) | 367 AA |
| 5 |  | [*Cortinarius obtusus*](https://www.uniprot.org/taxonomy/91495) | [K7R497](https://www.uniprot.org/uniprotkb/K7R497/entry) | 77 AA |
| 6 |  | [*Hericium erinaceus*](https://www.uniprot.org/taxonomy/91752) | [E2FB79](https://www.uniprot.org/uniprotkb/E2FB79/entry) | 359 AA |
| 7 |  | [*Lentinula edodes*](https://www.uniprot.org/taxonomy/5353) | [B5U990](https://www.uniprot.org/uniprotkb/B5U990/entry) | 376 AA |
| 8 |  | [*Phanerodontia chrysosporium*](https://www.uniprot.org/taxonomy/2822231) | [P19136](https://www.uniprot.org/uniprotkb/P19136/entry) | 382 AA |
| 9 |  | [*Phanerochaete sordida*](https://www.uniprot.org/taxonomy/48140) | [Q8NKA7](https://www.uniprot.org/uniprotkb/Q8NKA7/entry) | 382 AA |
| 10 |  | [*Phlebia radiata*](https://www.uniprot.org/taxonomy/5308) | [Q70LM3](https://www.uniprot.org/uniprotkb/Q70LM3/entry) | 390 AA |
| 11 |  | [*Pholiota nameko*](https://www.uniprot.org/taxonomy/61267) | [A0A120MUS1](https://www.uniprot.org/uniprotkb/A0A120MUS1/entry) | 371 AA |
| 12 |  | [*Pleurotus ostreatus*](https://www.uniprot.org/taxonomy/5322) | [O74179](https://www.uniprot.org/uniprotkb/O74179/entry) | 352 AA |
| 13 |  | [*Trametes pubescens*](https://www.uniprot.org/taxonomy/154538) | [A0A1M2V5W0](https://www.uniprot.org/uniprotkb/A0A1M2V5W0/entry) | 110 AA |
| 14 |  | [*Trametes versicolor*](https://www.uniprot.org/taxonomy/5325) | [Q99060](https://www.uniprot.org/uniprotkb/Q99060/entry) | 364 AA |

**Table S4.** Variance analysis of regression equation for ZEN degradation by the *C. cylindracea* SD-Cc78 culture supernatant.

| **Source** | **Sum of squares** | **df** | **Mean squares** | **F-value** | **P-value（Prob>F）** |  |
| --- | --- | --- | --- | --- | --- | --- |
| **Model** | 3693.06 | 9 | 410.34 | 672.19 | < 0.0001 | significant |
| A-Time | 234.34 | 1 | 234.34 | 383.89 | < 0.0001 |  |
| B-pH | 467.07 | 1 | 467.07 | 765.13 | < 0.0001 |  |
| C-Temperature | 46.06 | 1 | 46.06 | 75.46 | < 0.0001 |  |
| AB | 7.38 | 1 | 7.38 | 12.09 | 0.0103 |  |
| AC | 1.96 | 1 | 1.96 | 3.22 | 0.1160 |  |
| BC | 1.92 | 1 | 1.92 | 3.14 | 0.1198 |  |
| A² | 37.87 | 1 | 37.87 | 62.03 | 0.0001 |  |
| B² | 2634.84 | 1 | 2634.84 | 4316.24 | < 0.0001 |  |
| C² | 135.12 | 1 | 135.12 | 221.35 | < 0.0001 |  |
| **Residual** | 4.27 | 7 | 0.6104 |  |  |  |
| Lack of Fit | 2.60 | 3 | 0.8672 | 2.08 | 0.2462 | not significant |
| Pure Error | 1.67 | 4 | 0.4179 |  |  |  |
| **Cor Total** | 3697.34 | 16 |  |  |  |  |
| R^2^=0.9988, R_Adj_^2^=0.9974 | | | | | | |
